# Control of Conformational Transitions by the Conserved GX_9_P Motif in the Fifth Transmembrane Domain of Neurotransmitter Sodium Symporters

**DOI:** 10.3390/ijms26073054

**Published:** 2025-03-26

**Authors:** Xintong Zhang, Yanhong Xu, Qingyang Chen, Chan Li, Yuan-Wei Zhang

**Affiliations:** School of Life Sciences, Guangzhou University, Guangzhou 510006, China

**Keywords:** neurotransmitter sodium symporter, serotonin transporter, conformational transition, transport mechanism, TM5 unwinding, GX_9_P motif

## Abstract

The neurotransmitter sodium symporters (NSSs) play critical roles in the neurotransmission of monoamine and amino acid neurotransmitters and are the molecular targets of therapeutic agents in the treatment of several psychiatric disorders. Despite significant progress in characterizing structures and transport mechanisms, the management of conformational transitions by structural elements coupled with ion and substrate binding remains to be fully understood. In the present study, we biochemically identified a conserved GX_9_P motif in the fifth transmembrane domain (TM5) of the serotonin transporter (SERT) that plays a vital role in its transport function by facilitating conformational transitions. Mutations of the conserved Gly278 or Pro288 in the GX_9_P motif dramatically decreased specific transport activity by reducing the substrate binding-induced conformational transitions from an outward-open to an inward-open conformation. In addition, cysteine accessibility measurements demonstrated that the unwinding of the intracellular part of TM5 occurs during conformational transitions from an outward-open state, through an occluded state, to an inward-open state and that substrate binding triggers TM5 unwinding. Furthermore, mutations of the GX_9_P motif were shown to result in destructive effects on TM5 unwinding, suggesting that the GX_9_P motif controls conformational transitions through TM5 unwinding. Taken together, the present study provides new insights into the structural elements controlling conformational transitions in NSS transporters.

## 1. Introduction

The neurotransmitter sodium symporter (NSS) family comprises a group of transport proteins responsible for the reuptake of neurotransmitters, such as monoamines and amino acids, from the synaptic cleft after their release from presynaptic neurons and play critical roles by terminating neurotransmitter signaling in the central nervous system (CNS). The NSS members include the serotonin transporter (SERT), dopamine transporter (DAT), norepinephrine transporter (NET), γ-aminobutyric acid transporter (GAT), glycine transporter (GlyT), and so on. Notably, the dysfunction of these transporters is associated with a variety of neuropsychiatric disorders and, thereby, they are the molecular targets of many therapeutic agents used to treat depression, anxiety, attention deficit hyperactivity disorder, and schizophrenia [1,2,3,4].

The NSS transporters utilize transmembrane ion gradients of Na^+^ and Cl^−^ to drive their substrates across the membrane by symport with Na^+^ [5,6,7,8,9]. According to an alternating access mechanism, the central binding site is alternately accessible to the extracellular medium or cytoplasm for substrate binding or release by conformational transitions that open and close the extracellular or cytoplasmic substrate permeation pathways [10,11,12,13]. Because of the vital importance of conformational transitions in transport mechanisms, it becomes essential to understand how substrate, ion, and ligand binding events influence the conformation of NSS transporters.

The crystal structures of leucine transporter (LeuT), a bacterial member of the NSS transporters, at several conformational states have provided a good structural model to understand conformational transitions during substrate transport [14,15,16]. Biochemical analyses have demonstrated that Na^+^ binding, particularly at the Na2 site, stabilizes LeuT in an outward-open conformation and that substrate binding overcomes the effect of Na^+^, allowing conformational transitions to an inward-open state for substrate and Na^+^ release into the cytoplasm [14,15,17,18,19]. In addition, early studies indicated that intracellular K^+^ promotes SERT transport activity by K^+^ antiport [20,21]; however, it was not until recently that the K^+^ binding site was identified to be the Na2 site [22], indicating a pivotal role of the Na2 site in the arrangement of the sequential binding of Na^+^ and then K^+^ ions to facilitate serotonin (5-hydroxytryptamine, 5-HT) uptake by SERT. Subsequent studies of LeuT and DAT have demonstrated that K^+^ binding influences conformational transitions by inducing the closure of the extracellular pathway [23,24,25]. Most importantly, the recently resolved cryo-EM structures of NSS transporters including SERT, DAT, NET, GlyT1, and GAT1 have revealed that these transporters share a common LeuT fold and a similar conformational mechanism [26,27,28,29,30,31,32,33,34,35,36]. Despite significant progress in characterizing the structures and transport mechanisms of the NSS transporters, the management of conformational transitions by the structural elements coupled with ion and substrate binding remains to be fully addressed.

We previously demonstrated that the intracellular half of the fifth transmembrane domain (TM5) contributes to the cytoplasmic substrate permeation pathway in SERT [37]. The accessibility of this region was shown to depend on conformational transitions corresponding to the interconversion of SERT between the outward-open and inward-open states in response to Na^+^, substrate, or ligand binding [11,37]. Interestingly, structural and functional analyses for multi-hydrophobic amino acid transporter (MhsT), another bacterial member of the NSS transporters, have revealed that the partial unwinding of TM5 occurs in an inward-facing occluded conformation and that a conserved GlyX_9_Pro (GX_9_P) motif plays an important role in transport function [38]. Furthermore, the cryo-EM structures of SERT bound with the noncompetitive inhibitors ibogaine or noribogaine have indicated two TM5 unwinding regions, one in the middle of TM5 in an occluded conformation and another in the intracellular end of TM5 in an inward-open conformation [27]. However, these studies did not provide biochemical evidence to establish the significance of the GX_9_P motif in conformational transitions or the regulation of the TM5 unwinding by ion and substrate binding events in the transport process.

In the present study, we investigated the effects of the GX_9_P motif on TM5 unwinding and the conformational response to various binding events by using mutational and accessibility analyses for SERT. Our results indicated that the conserved GX_9_P motif plays a vital role in conformational transitions through control of TM5 unwinding. The present study provides new insights into the structural elements controlling conformational transitions of the NSS transporters in transport mechanisms.

## 2. Results

### 2.1. Sequence Alignment of the NSS Members and Structural Comparison of the GX_9_P Motif in SERT

**Figure 1:**
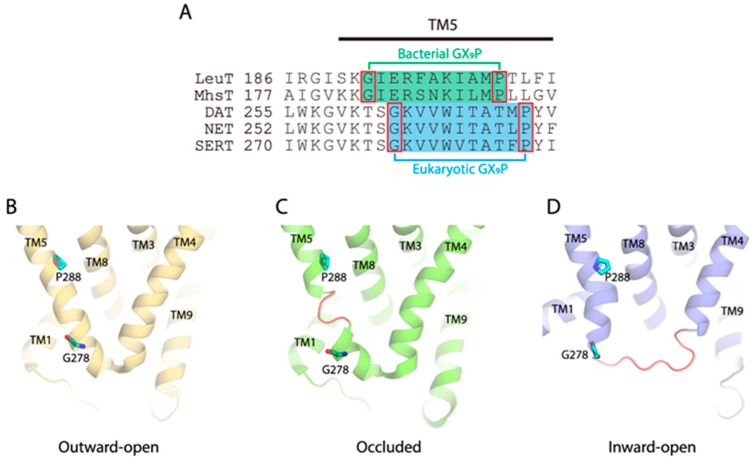
Amino acid sequence alignment of the NSS transporters and structural comparison of the GX_9_P motif in SERT. (**A**) Amino acid sequence alignment of the intracellular part of TM5 of several eukaryotic NSS transporters and bacterial members. Each line represents a different sequence with the residue number preceding the sequence. The GX_9_P motifs in bacterial and eukaryotic NSS transporters are highlighted in green and blue background, respectively. The conserved glycine and proline residues are marked by red rectangles. (**B**–**D**) Structural comparison of the GX_9_P motif of SERT in an outward-open ((**B**), PDB code, 6DZY), an occluded ((**C**), 6DZV), and an inward-open conformation ((**D**), 6DZZ). The conserved glycine and proline residues are colored cyan in each structure. The unwinding regions in TM5 are shown in red loops.

The GX_9_P motif (Gly278X_9_Pro288 in SERT) is completely conserved across the NSS family with a two-residue shift in eukaryotic NSS members with respect to bacterial members (Figure 1A). Strikingly, a sequence alignment with other Na^+^-dependent, LeuT-fold transporters showed that the characteristic GX_n_P pattern is also present in other transporter families, indicating the importance of the conserved motif in the transport mechanism [38,39]. Indeed, the helix-breaking GX_9_P motif leads to two populations for TM5 unwinding in conformational transitions of SERT [27]. One occurs between Val281 and Thr284 in the middle of TM5 when SERT is transformed from an outward-open to an occluded conformation, while another occurs at the cytoplasmic end of TM5 when SERT is converted from an occluded to an inward-open conformation (Figure 1B–D). The two unwinding events do not happen at the same time; rather, they are consecutive in conformational transitions from an outward-open state for Na^+^ and substrate binding, through an occluded state, to an inward-open state for Na^+^ and substrate release.

### 2.2. Mutational Analysis for the GX_9_P Motif

**Figure 2:**
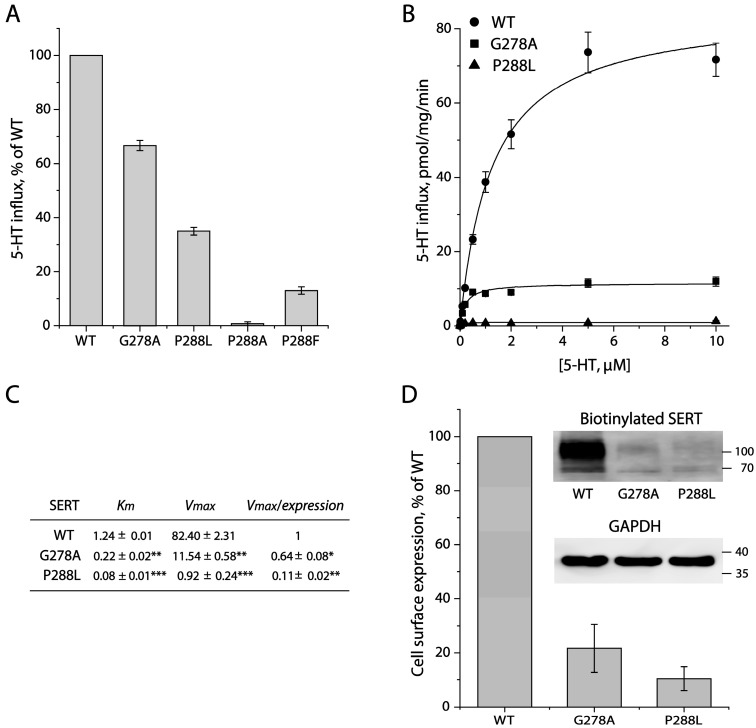
Functional analysis for the mutants of the GX_9_P motif. (**A**) Relative initial transport activity. 5-HT uptake was measured by incubation of the cells stably expressing SERT-WT or each GX_9_P mutant with 20 nM [^3^H]5-HT at 22 °C for 10 min, as described in Section 4. Transport activity was expressed as a percentage of the value obtained with SERT-WT. *n* = 3. (**B**) Kinetic analysis for SERT-WT, G278A, and P288L. Transport assay was performed by incubation of the cells stably expressing SERT-WT, G278A, or P288L with a range of 5-HT concentrations (0–10 μM), as described in Section 4. The graph shows a representative experiment with triplicate measurements at each 5-HT concentration. *n* = 3. (**C**) Kinetic parameters and specific transport activities of SERT-WT, G278A, and P288L. V_max_ and K_m_ values are presented as mean ± SEM (*n* = 3). Specific transport activity (V_max_/expression) is expressed as a ratio of V_max_ normalized to the cell surface expression relative to SERT-WT, and presented as mean ± SEM (*n* = 3). * *p* < 0.05, ** *p* < 0.01, *** *p* < 0.0001, compared to WT. (**D**) Cell surface expression of SERT-WT, G278A, and P288L. Biotinylation of SERT expression on the cell surface was performed as described in Section 4. Cell surface expression is expressed as a percentage of the integrated density of SERT-WT normalized to the internal GAPDH (*n* = 3). Insert shows representative immunoblots of biotinylated SERT and internal GAPDH.

To examine the functional role of the GX_9_P motif in SERT, we performed a mutational analysis for the conserved Gly278 and Pro288 residues. The replacement of Gly278 with Ala or Pro288 with Leu, Ala, or Phe significantly decreased the initial transport activity of SERT measured at a 20 nM [^3^H]5-HT concentration (Figure 2A). It is noticeable that substituting Pro288 with Ala or Phe led to more profound damage to its function, compared to that with the P288L mutation. Further kinetic analysis of the GX_9_P mutants showed that the mutation of Gly278 to Ala or Pro288 to Leu dramatically reduced the 5-HT uptake, with a V_max_ value of approximately 14% or 1% of that for WT, respectively, while the K_m_ was 5-fold, for G278A, or 15-fold, for P288L, lower than that for WT (Figure 2B,C), consistent with the observations reported for similar mutations in MhsT [38]. We then performed biotinylation experiments to determine the expression levels of the mutants on the cell surface. As shown in Figure 2D, the cell surface expression level of G278A or P288L normalized to the internal GAPDH was 21% or 10% of that of WT, respectively, indicating that these mutations also led to a significant reduction in SERT expression on the cell surface. Thus, the overall transport activity (V_max_) of G278A or P288L normalized to its cell surface expression (specific transport activity) was estimated to be decreased to only 64% or 11% of that for WT, respectively (Figure 2C), suggesting that mutations of the GX_9_P motif significantly impaired the specific transport activity but increased the substrate binding affinity (K_m_).

### 2.3. Effects of the GX_9_P Mutations on SERT Conformation

**Figure 3:**
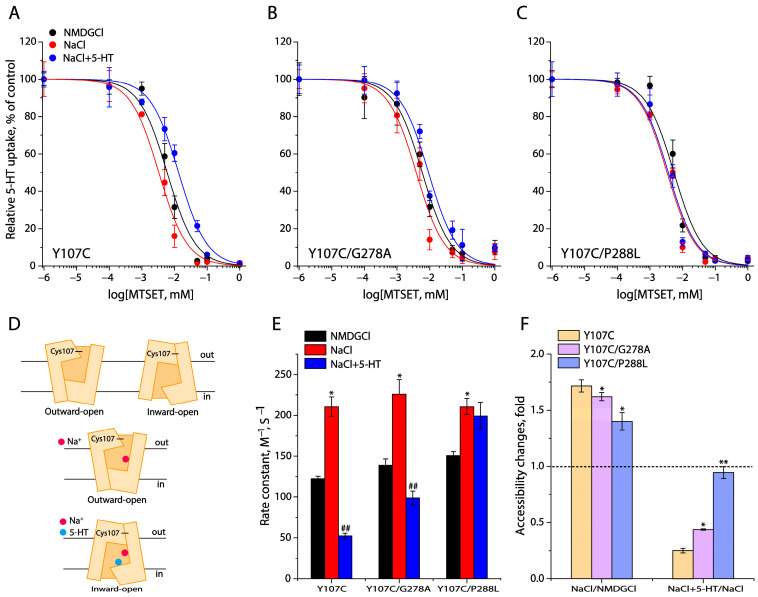
Effects of the GX_9_P mutations on conformation of the extracellular pathway in SERT. (**A**) MTSET concentration-dependent inhibition of 5-HT uptake by Y107C/C109A. (**B**) MTSET concentration-dependent inhibition of 5-HT uptake by Y107C/C109A/G278A. (**C**) MTSET concentration-dependent inhibition of 5-HT uptake by Y107C/C109A/P288L. In (**A**–**C**), MTSET inhibition was parallelly examined under different conditions, such as NMDGCl (control), NaCl, or 5-HT/NaCl, as described in Section 4. The graphs show representative experiments for each mutant, respectively. *n* = 3. (**D**) Schematic presentation of Cys107 positions in response to different conformations of SERT. Cl^−^ ion was set to bind to the Cl^−^ site in all conformational states. Upper panel shows SERT under the control conditions with NMDGCl, in which SERT presents in a dynamic equilibrium between the outward-open and inward-open conformational states, while middle and lower panels represent SERT under NaCl and 5-HT/NaCl, respectively. (**E**) Rate constants for the reactivities with MTSET. The MTSET concentration giving half-maximal inhibition of 5-HT uptake in the cells (**A**–**C**) was used to calculate rate constant for the reactivity with MTSET. Error bars represent ± SEM. *n* = 3. * *p* < 0.05, ** *p* < 0.01, compared to the corresponding control rate constant obtained with NMDGCl. ^##^ *p* < 0.01, compared to the corresponding rate constant obtained with NaCl. (**F**) Accessibility change of each indicated mutant between two conditions, expressed as ratios of rate constants, such as a ratio of rate constants under NaCl over NMDGCl or a ratio of rate constants under 5-HT+NaCl over NaCl. The dotted line shows no accessibility change between two conditions (accessibility change fold is 1). * *p* < 0.05, ** *p* < 0.01, compared to Y107C (*n* = 3).

To investigate the effects of the GX_9_P mutations on SERT conformation, we performed cysteine accessibility measurements, according to an approach to evaluate SERT conformations under various conditions in the extracellular permeation pathway [40,41]. The cysteine mutant employed for this study was Y107C in an extracellular cysteine-less background C109A, in which a cysteine residue was strategically positioned in the extracellular pathway (Figure 3A,D). As shown in Figure 3A, the 5-HT uptake by Y107C was significantly inhibited by preincubation with a membrane-impermeant reagent, 2-(trimethylammonium) ethyl methanethiosulfonate bromide (MTSET). The MTSET concentration-dependent inhibition was sensitive to Na^+^ binding or 5-HT transport in the presence of both Na^+^ and Cl^−^. Specifically, Na^+^ binding stabilized an outward-open conformation of SERT (Figure 3D), which led to the exposure of Cys107 to aqueous MTSET and, subsequently, a faster MTSET inhibition of 5-HT uptake (Figure 3A) and a higher rate constant for the reactivity with MTSET than those for the control (NMDGCl, N-methyl-D-glucamine chloride) (Figure 3E). In contrast, 5-HT transport in the presence of both Na^+^ and Cl^−^ ions stabilized an inward-open conformation of SERT (Figure 3D), which resulted in the closure of the extracellular pathway and thereby a slower MTSET inhibition of 5-HT uptake and a lower rate constant for the reactivity with MTSET than those for the control (Figure 3A,E).

Mutations of the GX_9_P residues Gly278 to Ala or Pro288 to Leu resulted in alterations in the accessibility of Y107C to MTSET in response to Na^+^ binding or 5-HT transport. Although the Y107C/G278A and Y107C/P288L mutants showed a conformational response to Na^+^ binding (Figure 3B,C,E), the Na^+^-induced accessibility increases were smaller than that for the background Y107C (Figure 3F). Specifically, the G278A mutation led to a small but significant attenuation (the ratio of rate constants under NaCl over NMDGCl, 1.72 for Y107C vs. 1.62 for G278A/Y107C) of the Na^+^-induced accessibility increase, whereas the P288L mutation produced a profound reduction in the Na^+^-induced accessibility increase (the ratio of rate constants under NaCl over NMDGCl, 1.72 for Y107C vs. 1.40 for P288L/Y107C). In addition, Y107C/G278A showed an accessibility decrease in response to the incubation of 5-HT in the presence of both Na^+^ and Cl^−^, the decrease in accessibility, however, was less than that for its background Y107C (Figure 3E,F). Furthermore, the accessibility of Y107C/P288L was no longer sensitive to the incubation of 5-HT in the presence of both Na^+^ and Cl^−^ (Figure 3E,F), consistent with a dramatic loss of its ability to transport 5-HT (Table 1). These results suggest that such mutations of the GX_9_P motif led to the stabilization of an outward-open conformation of SERT to different extents, which promotes the substrate binding affinity but impairs substrate transport by alleviating conformational transitions to an inward-open state.

### 2.4. Conformational Analysis for Unwinding in the Middle of TM5

**Figure 4:**
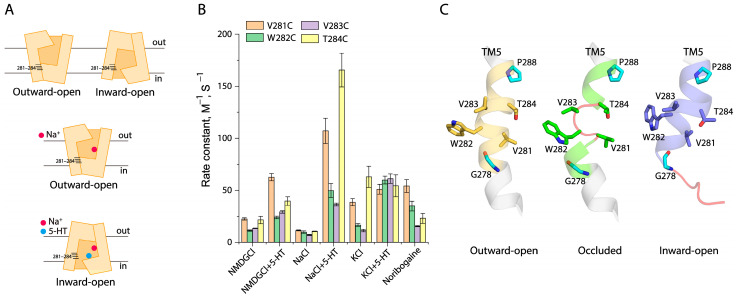
Conformational analysis for the unwinding of the middle of TM5. (**A**) Schematic presentation of positions of the residues 281–284 in the middle of TM5 in response to different conformations of SERT. A Cl^−^ ion was set to bind to the Cl^−^ site in all conformational states. The upper panel shows SERT under the control conditions with NMDGCl, in which SERT presents in a dynamic equilibrium between the outward-open and inward-open conformational states, while the middle and lower panels represent SERT under NaCl and 5-HT/NaCl, respectively. (**B**) Rate constants of cysteine mutants in the middle of TM5 under various ion and substrate binding conditions. MTSEA concentration-dependent inhibition of ASP^+^ binding was performed as described in Section 4 (Figures S1 and S2). The MTSEA concentration giving half-maximal inhibition of ASP^+^ binding was used to calculate rate constant for the reactivity with MTSEA. Error bars represent ±SEM (*n* = 3). (**C**) Structural comparison of the unwinding region in the middle of TM5 in an outward-open (PDB code, 6DZY), an occluded (6DZV), and an inward-open (6DZZ) conformation. The conserved glycine and proline residues are colored cyan in each structure. The unwinding region in the middle of TM5 is shown in a red loop.

The structural analysis indicated that the TM5 unwinding occurred between residues 281 to 284 in an occluded conformation of SERT (Figure 4A,C). To investigate the effects of ion or substrate binding on the TM5 conformation, we performed a conformational analysis through cysteine accessibility measurements in the cytoplasmic pathway [40,42]. Each residue from the region of residues 281 to 284 in TM5 was mutated to cysteine, one at a time, in a cysteine-less background (X5C), respectively, and these cysteine mutants were then employed to examine their accessibility to 2-aminoethyl methanethiosulfonate hydrobromide (MTSEA) in digitonin-permeabilized cells [40]. From the MTSEA concentration-dependent inhibition of ligand (ASP^+^) binding (Figures S1 and S2), we calculated the rate constants of the reactivities of these cysteine mutants with MTSEA, which were used to assess their accessibility under various ion or substrate binding events (Figure 4B). In the control (NMDGCl), SERT is believed to be present in a dynamic equilibrium among various conformational states (Figure 4A, upper panel). Our accessibility results showed that V281C and T284C were slightly more accessible than other two mutants, W282C and V283C (Figure 4B), consistent with the fact that Val281 and Thr284 face the cytoplasmic pathway while the other two residues are located on the inaccessible side of the TM5 α-helix. Co-incubation with substrate 5-HT and NMDGCl led to increases in the MTSEA accessibility of all of the cysteine mutants but did not alter its α-helical feature in the 281–284 region (Figure 4B). In contrast, because Na^+^ stabilized SERT in an outward-open (inward-closed) conformation (Figure 4A, middle panel), all of the four cysteine mutants were inaccessible from the cytoplasm (Figure 4B). On the other hand, 5-HT transport induced SERT to an inward-open conformation (Figure 4A, lower panel), all mutants, therefore, showed dramatic increases in cytoplasmic accessibility (Figure 4B). Consistent with the SERT structures, our accessibility measurements of these cysteine mutants suggest that this region is in an α-helical conformation in an inward-open state induced by 5-HT in the presence of both Na^+^ and Cl^−^ (Figure 4B,C).

In agreement with the cryo-EM structures that revealed SERT in an inward-open conformation in the presence of KCl [43], our cytoplasmic accessibility measurements indicated K^+^ binding stabilized an intermediate conformational state of SERT, in which the cytoplasmic pathway partially opens to the cytoplasm with the 281–284 region retained in an α-helical pattern, similar to that in the fully inward-open state induced by 5-HT transport (Figure 4B). On the other hand, the incubation of substrate 5-HT in the presence of KCl dramatically increased the MTSEA accessibility of W282C and V283C to the levels of V281C and T284C, indicating that substrate binding in the presence of KCl induced unwinding of the 281–284 region in the middle of TM5 (Figure 4B). Furthermore, the binding of a substrate analogue, noribogaine, at the central binding site has been shown to close the extracellular pathway and subsequently open the cytoplasmic pathway in the cryo-EM structures of SERT–ibogaine complexes [27]. Our accessibility results indicated that noribogaine binding resulted in alterations in the accessibility pattern of the 281–284 region, which tends to be in an unwinding conformation (Figure 4B). These results suggest that the substrate or its analogue coupled with K^+^ or Na^+^ binding is essential for unwinding in the middle of TM5 and promotes conformational transitions from an outward-open to an occluded conformational state (Figure 4C).

### 2.5. Effects of the GX_9_P Mutations on Unwinding in the Middle of TM5

**Figure 5:**
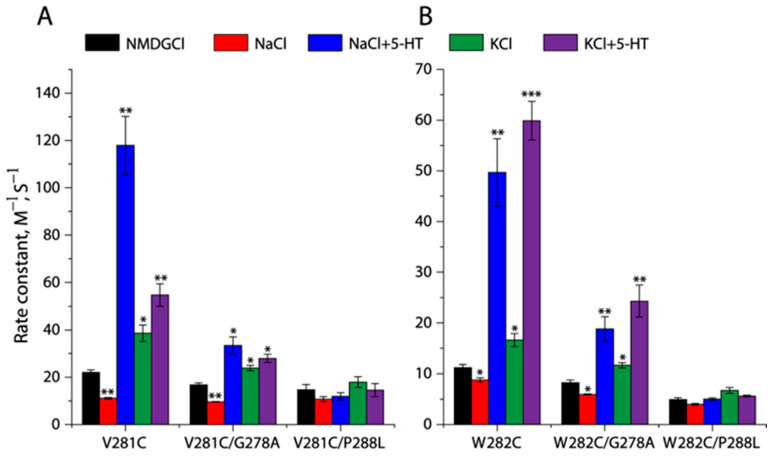
Effects of the GX_9_P mutations on unwinding in the middle of TM5. Effects of G278A or P288L on rate constants for the reactivities with MTSEA of V281C (**A**) or W282C (**B**) under various ion and substrate binding conditions were examined by measuring inhibition of ASP^+^ binding by MTSEA over a range of concentrations (Figure S3). MTSEA concentration-dependent inhibition of ASP^+^ binding was determined as described in Section 4. The MTSEA concentration causing half-maximal inhibition of ASP^+^ binding was used to calculate rate constant for the reactivity with MTSEA. Error bars represent ±SEM (*n* = 3). * *p* < 0.05, ** *p* < 0.01, *** *p* < 0.001, compared to the corresponding control rate constant obtained with NMDGCl.

To investigate the effects of the GX_9_P motif on TM5 unwinding, we introduced mutations of the conserved GX_9_P residues, Gly278 to Ala or Pro288 to Leu, into the two representative cysteine mutants, V281C and W282C, respectively, in the TM5 unwinding region and examined their effects on the cytoplasmic MTSEA accessibility (Figure S3). As shown in Figure 5A,B, compared to the corresponding background, V281C or W282C, the mutation of Gly278 to Ala remarkably decreased the rate constants for the reactivity of V281C or W282C with MTSEA but still retained sensitivity to various ion or substrate binding events. In contrast, the mutation of Pro288 to Leu led to a profound reduction in the MTSEA accessibility of V281C or W282C that was no longer responsive to various binding events (Figure 5A,B), suggesting that SERT was unable to facilitate the unwinding of the 281–284 region by the Pro288 to Leu mutation. In addition, as shown in Table 1, the effects of the GX_9_P mutations on the accessibility and transport function of these mutants are consistent.

### 2.6. Conformational Analysis for Unwinding of the Intracellular End of TM5

**Figure 6:**
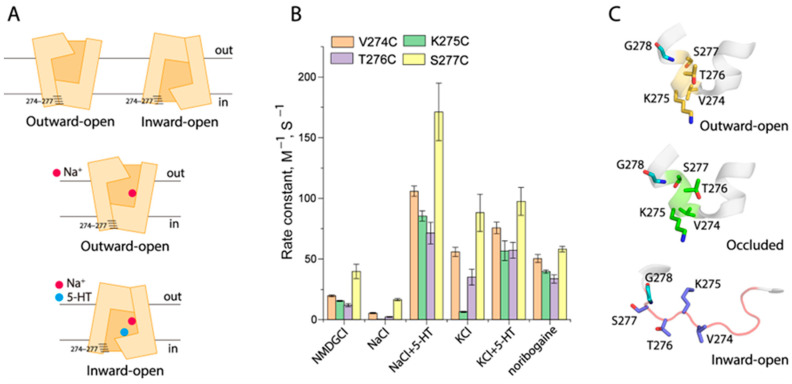
Conformational analysis for unwinding at the intracellular end of TM5. (**A**) Schematic presentation of positions of residues 274–277 at the intracellular end of TM5 in response to different conformations of SERT. Cl^−^ ion was set to bind to the Cl^−^ site in all conformational states. Upper panel shows SERT under the control conditions with NMDGCl, in which SERT presents in a dynamic equilibrium between the outward-open and inward-open conformational states, while middle and lower panels represent SERT under NaCl and 5-HT/NaCl, respectively. (**B**) Rate constants of cysteine mutants at the intracellular end of TM5 under various ion and substrate binding conditions. MTSEA concentration-dependent inhibition of ASP^+^ binding was determined as described in Section 4 (Figure S4). The MTSEA concentration causing half-maximal inhibition of ASP^+^ binding was used to calculate the rate constant for the reactivity with MTSEA. Error bars represent ±SEM (*n* = 3). (**C**) Structural comparison of the intracellular end of TM5 in an outward-open (PDB code, 6DZY), an occluded (6DZV), and an inward-open (6DZZ) conformation. The conserved glycine residue is colored cyan in each structure. The unwinding region in the intracellular end of TM5 is shown in a red loop.

A structural comparison revealed that more unwinding occurs at the intracellular end of TM5 in an inward-open conformation (Figure 6A,C). To investigate conformational changes in this region in response to ion or substrate binding, we carried out cytoplasmic accessibility measurements of several representative cysteine mutants including V274C, K275C, T276C, and S277C (Figure 6A and Figure S4). As shown in Figure 6B, compared to the control (NMDGCl), Na^+^ binding decreased the rate constants for the reactivities of these cysteine mutants with MTSEA due to the closure of the cytoplasmic pathway, whereas 5-HT transport increased them in order to open the pathway for substrate release. As mentioned above, SERT favors a partially inward-open conformation under KCl, consistent with our cytoplasmic accessibility results that showed the 274–277 region in a typical α-helical pattern (Figure 6B). In contrast, 5-HT or noribogaine binding in the presence of KCl or NaCl dramatically increased the MTSEA accessibility of K275C and T276C, resulting in an accessibility pattern that tended to unwind this region at the intracellular end of TM5, similar to that measured under 5-HT binding in the presence of NaCl. However, the rate constants for the reactivities with MTSEA of these cysteine mutants in a fully inward-open conformation induced by 5-HT/NaCl are much higher than those measured with 5-HT/KCl or noribogaine/NaCl binding, suggesting that SERT adopts a partially inward-open conformation with 5-HT or noribogaine binding in the presence of KCl or NaCl.

### 2.7. Effects of the GX_9_P Mutations on Unwinding at the Intracellular End of TM5

**Figure 7:**
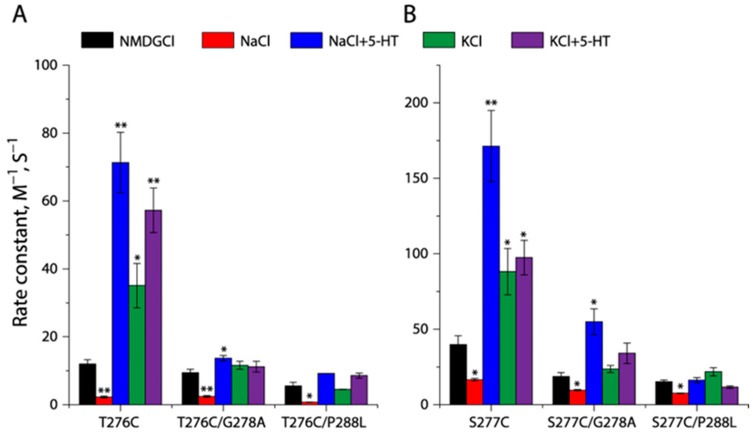
Effects of the GX_9_P mutations on unwinding at the intracellular end of TM5. Effects of G278A or P288L on rate constants for the reactivities of T276C (**A**) or S277C (**B**) with MTSEA under various ion and substrate binding conditions were examined, respectively. MTSEA concentration-dependent inhibition of ASP^+^ binding was determined as described in Section 4 (Figure S5). The MTSEA concentration giving half-maximal inhibition of ASP^+^ binding was used to calculate rate constant for reactivity with MTSEA. Error bars represent ±SEM (*n* = 3). * *p* < 0.05, ** *p* < 0.01, compared to the corresponding control rate constant obtained with NMDGCl.

To investigate the effects of GX_9_P mutations on the conformation of the intracellular end of TM5, we introduced the Gly278 to Ala or Pro288 to Leu mutation into two representative cysteine mutants, T276C and S277C (Figure S5). As shown in Figure 7A,B, the accessibility of both the T276C and S277C mutants were highly responsive to various binding events; mutating either Gly278 to Ala or Pro288 to Leu, however, dramatically reduced their ability to open the cytoplasmic pathway in response to KCl, 5-HT/KCl, or 5-HT/NaCl. Specifically, the Gly278 to Ala mutation led either T276C or S277C to a loss of sensitivity to MTSEA modification in KCl or 5-HT/KCl, while the Pro288 to Leu mutation produced a more disastrous effect on the cysteine mutants that did not even show an accessibility response to 5-HT/NaCl, consistent with the remarkable decreases in their transport activities (Table 1). In contrast, the GX_9_P mutations did not alter the sensitivity of these cysteine mutants to Na^+^ binding, which caused the closure of the cytoplasmic pathway and thereby reduced the MTSEA accessibility of these cysteine mutants (Figure 7A,B).

## 3. Discussion

The present study identified a GX_9_P motif in TM5 that plays a vital role in the transport function of SERT by using biochemical approaches. Our results indicated that mutations of the conserved Gly278 or Pro288 dramatically decreased specific transport activity by reducing conformational transitions from an outward-open to an inward-open state, and by blocking the occurrence of TM5 unwinding in response to ion and substrate binding in the transport process. These results support the proposal that the GX_9_P motif controls TM5 unwinding, essential for conformational transitions during 5-HT transport by SERT.

Consistent with the cryo-EM structures of SERT, our cysteine accessibility measurements demonstrated that TM5 unwinding occurs during conformational transitions from an outward-open to an inward-open state and that substrate binding triggers TM5 unwinding in the transport process. We previously identified a network of interactions involving Tyr108, Na1, and the substrate carboxyl group that triggers an interaction between the scaffold and bundle domains of LeuT, thereby enforcing the closure of the extracellular pathway and allowing conformational transitions from an outward-open to an inward-open conformation for substrate release into the cytoplasm [13]. Due to the lack of the carboxyl group of amino acid substrates, instead, SERT was assumed to form an interaction network with the carboxyl group of a conserved aspartate (Asp98) that is located between the TM3 tyrosine (Tyr179) and Na1 in the SERT structures, like the substrate carboxyl group in LeuT [26,44,45]. The formation of such an interaction network in either LeuT or SERT depends on substrate binding. This substrate-dependent effect on conformational transitions has also been identified in other NSS transporters [8,17,18,37,46,47,48]. Therefore, we suggest that all members in the NSS family share general features of the conformational mechanism, involving the facilitation of TM5 unwinding by the GX_9_P motif for substrate binding-induced conformational transitions.

**Figure 8:**
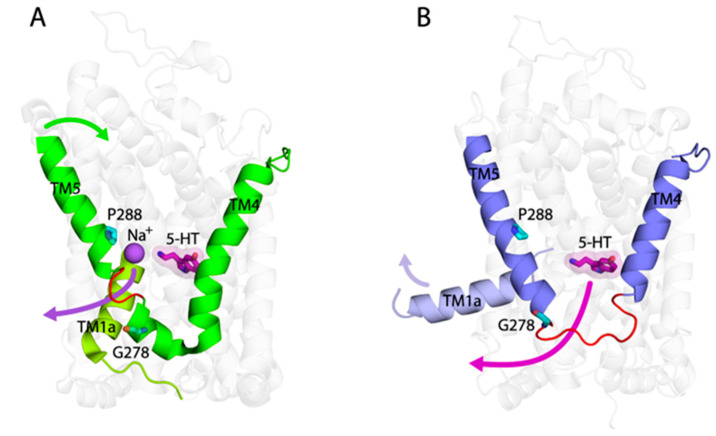
The proposed structural and functional role of TM5 in 5-HT transport by SERT. SERT is in an occluded (**A**) or inward-open (**B**) conformational state, respectively. The conserved glycine and proline residues are colored cyan in each structure. The unwinding regions in TM5 are shown in red loops. Arrows in green or purple in (**A**) show TM5 or Na^+^ (Na2) movement, while arrows in blue or purple in (**B**) show TM1a or 5-HT movement, respectively.

The structures of SERT indicated that the intracellular half of TM5 was unwound in two regions, in response to an occluded or an inward-open conformational state, respectively [27]. Our cytoplasmic cysteine accessibility measurements suggest two-step TM5 unwinding during substrate binding-induced conformational transitions from an outward-open, through an occluded state, to an inward-open state. In contrast, the unwinding of the entire intracellular half of TM5 in the substrate-bound MhsT was observed in an inward-facing occluded state [38]. TM5 unwinding in MhsT has been proposed to result from the combined strains imposed by the movement of the extracellular part of TM5 during the closure of the extracellular pathway and by the interactions between the intracellular part of TM5 and TM1a, also creating a solvent pathway for Na^+^ release from the Na2 site to the cytoplasm [38]. Although the unwinding region of TM5 in SERT is relatively shorter than that in MhsT, it has been proposed that this unwinding together with the shift of TM5 towards the membrane enables Na2 to access the cytoplasm [27] (Figure 8A). On the other hand, when SERT transitions from an occluded to an inward-open state, TM1a shifts away from TM5, resulting in a dramatic reduction in the interactions between the intracellular part of TM5 and TM1a and the subsequent restoration of the α-helical structure of the region with residues 281–284 accompanied by the occurrence of the unwinding of the intracellular end of TM5 for substrate release (Figure 8B). The different pattern of TM5 unwinding between the NSS transporters might be caused by the nature of each individual transporter. For example, like LeuT, MhsT is a transporter for hydrophobic L-amino acids and is thought to utilize the carboxyl group of amino acid substrates with Na1 and other residues to form an interaction network for enforcing the closure of the extracellular pathway, which then forces the intracellular part of TM5 to be unwound. However, SERT is a monoamine transporter and must use a carboxyl group supplied by the protein for the closure of the extracellular pathway, thereby leading to a variation in the MhsT mechanism to impose strain on TM5 that might produce a different pattern of TM5 unwinding. Taken together, TM5 unwinding not only is a consequence of substrate binding-induced structural rearrangements of TM5 for conformational transitions but also plays a functional role in Na^+^ ion and substrate release (Figure 8).

Most importantly, the present study uncovered a novel conformational mechanism by which the GX_9_P motif manages substrate binding-induced conformational transitions by controlling TM5 unwinding in the NSS transporters. Our results indicated that the Pro288 mutation led to a more profound effect on the conformational sensitivity of SERT to the substrate binding-induced closure of the extracellular pathway and TM5 unwinding, compared to that caused by the Gly278 mutation, suggesting that the helix-breaking residue proline in the GX_9_P motif in the middle of TM5 plays a central role in TM5 unwinding. Because the GX_9_P motif is completely conserved in the NSS family, we expected that this motif, especially the conserved proline residue, would be critical for its transport function through the facilitation of conformational transitions during the transport process in all NSS members.

Previously, we demonstrated that 5-HT transport promotes the PKG-mediated phosphorylation of Thr276, a residue located at the intracellular end of TM5, which, in turn, further increases the transport activity of SERT [49]. The present study shows that 5-HT transport induces TM5 unwinding, especially at the intracellular end region, which includes Thr276. Thus, it is most likely that 5-HT transport stabilizes SERT in an inward-open conformation, which leads to TM5 unwinding and allows the region containing Thr276 to extend into the cytoplasm, where it can be phosphorylated. On the other hand, because the intracellular part of TM5 lines the cytoplasmic substrate permeation pathway, which alternately opens and closes during the transport process, we assume that its modification, such as the phosphorylation of Thr276, could also alter the conformational dynamics of SERT, thereby stimulating its transport activity.

The present study aims to provide direct biochemical evidence for elucidating the role of the GX_9_P motif in conformational transitions by managing TM5 unwinding in SERT under various ion and substrate binding events. However, it is extremely difficult to exactly mimic the conformation of an intermediate state, such as an occluded state, under a biochemical experiment setting. Hence, further studies of the structure and dynamics are required for a comprehensive understanding of the role of GX_9_P motif-controlled TM5 unwinding in individual conformational states as well as conformational transitions in the transport process.

## 4. Materials and Methods

### 4.1. Materials

HeLa (CCL-2) and HEK 293T cells were from the American Type Culture Collection. [^3^H]5-HT (27.1 Ci/mmol) was purchased from PerkinElmer Life Sciences (Shelton, CT, USA). Monoclonal anti-FLAG M2 antibody, 3 x FLAG peptide, ASP^+^, 5-HT, and noribogaine were obtained from Sigma-Aldrich (St. Louis, MO, USA). Sulfosuccinimidyl 2-(biotinamido) ethyl-1,3-dithio propionate (sulfo-NHS-SS-biotin), streptavidin agarose gel, and Super Signal West Pico were from ThermoFisher Scientific (Waltham, MA, USA). MTSET and MTSEA were purchased from Biotium (Fremont, CA, USA). All other reagents were of analytical grade.

### 4.2. Mutagenesis and Stable Cell Line Generation

The lentiviral plasmids (Lenti-EF-1α-SERT-BSD, Lenti-EF-1α-C109A-BSD, lenti-EF-1α-X5C-BSD) encoding C-terminal FLAG-tagged SERT were used as templates for mutagenesis. All mutants were generated using the Mut Express II Fast Mutagenesis Kit (Vazyme, Nanjing, China) and confirmed by full-length DNA sequencing.

The lentivirus was prepared by transfecting a mixture of the lentiviral plasmid and two other packaging vectors, psPAX2 and pMD2G, into HEK 293T cells, as described previously [50]. HeLa cells were then infected by the lentivirus with polybrene in Dulbecco’s Modified Eagle’s Medium (DMEM) with blasticidin S at a concentration of 12 μg/mL. The culture medium was changed every three days until colonies of blasticidin S-resistant cells were formed. The cells were then maintained in DMEM supplemented with 10% fetal bovine serum, 100 units/mL penicillin, 100 μg/mL streptomycin, and 12 μg/mL blasticidin S at 37 °C in a humidified 5% CO_2_ incubator. The stable cell lines expressing SERT or its mutants were confirmed by 5-HT uptake assay and immunoblot analysis.

### 4.3. 5-HT Transport Assay

[^3^H]5-HT uptake was assayed with HeLa cells stably expressing SERT or its mutants at 22 °C. HeLa cells grown in 96-well plates with 100% confluence were washed once with 100 μL of KRH buffer (20 mM HEPES, pH 7.4, 120 mM NaCl, 1.3 mM KCl, 2.2 mM CaCl_2_, 1.2 mM MgSO_4_, and 0.1% glucose) and incubated in KRH buffer for 10 min at 22 °C with or without the indicated reagents. 5-HT uptake was initiated by addition of [^3^H]5-HT (20 nM final concentration), and the incubation was continued for 10 min. The assays were terminated by three rapid washes with ice-cold phosphate-buffered saline (PBS buffer, 137 mM NaCl, 2.7 mM KCl, 4.3 mM Na_2_HPO_4_, and 1.4 mM KH_2_PO_4_, pH 7.4). The cells were then solubilized in 30 μL of 0.1 M NaOH for 30 min. The extent of [^3^H]5-HT accumulated was determined by liquid scintillation spectrometry in a PerkinElmer Microbeta2 plate counter (Shelton, CT, USA). For kinetic analysis, cells were incubated with [^3^H]5-HT (kept at 20 nM) together with unlabeled 5-HT at a range of total 5-HT concentrations from 20 nM to 10 μM. All transport measurements were corrected by subtracting blank values measured in the presence of 100 μM fluoxetine. Protein concentration was determined with the Micro BCA protein assay reagent kit (ThermoFisher Scientific, Waltham, MA, USA).

### 4.4. Cell Surface Biotinylation

Cell surface expression of SERT WT or mutants was determined using the membrane-impermeant biotinylation reagent sulfo-NHS-SS-biotin as described previously [51]. In brief, HeLa cells stably expressing SERT WT or mutants were treated twice with sulfo-NHS-SS-biotin for 20 min on ice. After rinsing with 100 mM glycine in PBS for 20 min on ice to quench excess sulfo-NHS-SS-biotin, the cells were lysed, and the biotinylated proteins were captured by streptavidin-agarose beads in an overnight incubation at 4 °C with gentle agitation. The biotinylated proteins were eluted with 100 μL of SDS-PAGE sample buffer and separated on a 10% SDS-polyacrylamide gel. The transporters were visualized by Western blot with monoclonal anti-FLAG M2 antibody (1:1000) against the FLAG epitope tag at the C-terminus of SERT. The amount of cell surface expression was determined by quantitative luminescence imaging using an eBlot touch imager (Shanghai, China).

### 4.5. Cysteine Accessibility Measurements

Cysteine accessibility in the extracellular pathway was measured using HeLa cells stably expressing Y107C/109A, which was used as an indicator for the cysteine accessibility changes in response to ion or ligand binding, as described previously [42]. In brief, the cells in poly-D-lysine-precoated 96-well plates were incubated with a membrane-impermeant cysteine reagent MTSET over a range of concentrations for 15 min at 22 °C in HEPES buffer (25 mM HEPES, pH 7.5, adjusted by LiOH) containing 150 mM of the indicated ions with or without 10 μM 5-HT. After washing three times with the same buffer to quench residual substrate and unreacted MTSET, 5-HT transport assay was performed by incubating the cells with 20 nM [^3^H]5-HT in KRH buffer for an additional 10 min at 22 °C, as described above.

Cysteine accessibility in the cytoplasmic pathway was measured with digitonin-permeabilized HeLa cells stably expressing each individual cysteine mutant, in which the indicated position was replaced by cysteine, according to a protocol described previously [40]. The cells grown in poly-D-lysine-precoated 24-well plates were incubated with MTSEA at a range of concentrations in HEPES buffer containing 150 mM of the indicated ions with or without 10 μM 5-HT or noribogaine in the presence of 25 μg/mL digitonin at 22 °C for 15 min. After washing free of unreacted MTSEA and ligands, the cells were incubated with 10 μM ASP^+^ in KRH buffer at 22 °C for an additional 5 min. Excessive ASP^+^ was removed by 3 x rapid washing with KRH buffer and ASP^+^ fluorescence retained in the cell membrane was measured by fluorescence spectrometry with an Infinite 200 Pro microplate reader (Tecan, Männedorf, Switzerland). Non-specific ASP^+^ binding was determined by adding 100 μM fluoxetine in the incubation mixture and used to correct ASP^+^ binding measurements.

The MTSET or MTSEA concentration causing half-maximal inactivation of 5-HT uptake for the extracellular accessibility measurements or ASP^+^ binding for the cytoplasmic accessibility measurements was determined and used to calculate the rate constant for cysteine reactivity, respectively, as described previously [40]. These accessibility measurements depend on the ability of MTSET or MTSEA to inactivate substrate transport or ligand binding activity by an allosteric mechanism [52].

### 4.6. Data Analysis

Nonlinear regression fits of experimental and calculated data were performed with Origin 2021 version 9.8.0.200 (Origin Lab, Northampton, MA, USA). The statistical analysis was from multiple experiments. Data with error bars represent the mean ± standard error of mean (SEM) for at least three independent experiments. Statistical analysis was performed using one-way ANOVA followed by post hoc tests, and statistical significance was set at *p* < 0.05.

## 5. Conclusions

The present study characterized a conserved GX_9_P motif in the management of conformational transitions through controlling TM5 unwinding in SERT by using biochemical approaches. Our results indicated that mutations of Gly278 or Pro288 in the GX_9_P motif significantly decreased the transport activity by decreasing substrate binding-induced conformational transitions in SERT. In addition, the cysteine accessibility measurements demonstrated that the unwinding of the intracellular part of TM5 occurs during conformational transitions from an outward-open state, through an occluded state, to an inward-open state, and that mutations of Gly278 or Pro288 led to destructive effects on TM5 unwinding. These observations suggest that the GX_9_P motif plays a vital role in the transport function by facilitating conformational transitions through TM5 unwinding.

## Figures and Tables

**Table 1:** Relative transport activity of SERT mutants in TM5.

Background	Mutants	Transport Activity (% of WT)
hSERT WT		100
C109A/Y107C		69.32 ± 6.28
	C109A/Y107C/G278A	40.27 ± 7.63 **
	C109A/Y107C/P288L	9.79 ± 2.60 ***
X5C		74.53 ± 12.23
	V281C	80.04 ± 3.23
	W282C	75.01 ± 2.71
	V283C	76.26 ± 2.26
	T284C	78.06 ± 1.00
	V281C/G278A	48.60 ± 1.53 **
	V281C/P288L	11.98 ± 3.19 ***
	W282C/G278A	8.51 ± 1.07 ***
	W282C/P288L	1.12 ± 1.13 ***
	V274C	74.08 ± 2.14
	K275C	75.28 ± 1.89
	T276C	70.46 ± 2.28
	S277C	72.38 ± 1.66
	T276C/G278A	6.62 ± 1.70 ***
	T276C/288L	0.56 ± 0.46 ***
	S277C/G278A	59.27 ± 2.07 *
	S277C/P288L	13.20 ± 0.69 ***

5-HT uptake was measured by incubating the cells stably expressing SERT-WT or each mutant with 1 μM 5-HT at 22 °C for 10 min, as described in Section 4. Transport activity was expressed as a percentage of that measured with SERT-WT. The experiments were performed 3 times (*n* = 3) with triplicate measurements for each mutant in each experiment. * *p* < 0.05; ** *p* < 0.01; *** *p* < 0.001, compared to WT.

## Data Availability

The data presented in this study are available on request from the corresponding author.

## Supplementary Materials

The following supporting information can be downloaded at www.mdpi.com/article/10.3390/ijms26073054/s1.
