# Supplementary material for: Control of Conformational Transitions by the Conserved GX9P Motif in the Fifth Transmembrane Domain of Neurotransmitter Sodium Symporters
Source: Int J Mol Sci. 2025 Mar 26;26(7):3054. doi: 10.3390/ijms26073054 (PMC11988846; doi:10.3390/ijms26073054)
Supplement: Supplementary file 1 [file ijms-26-03054-s001.zip › ijms-3532763-supplementary.pdf]

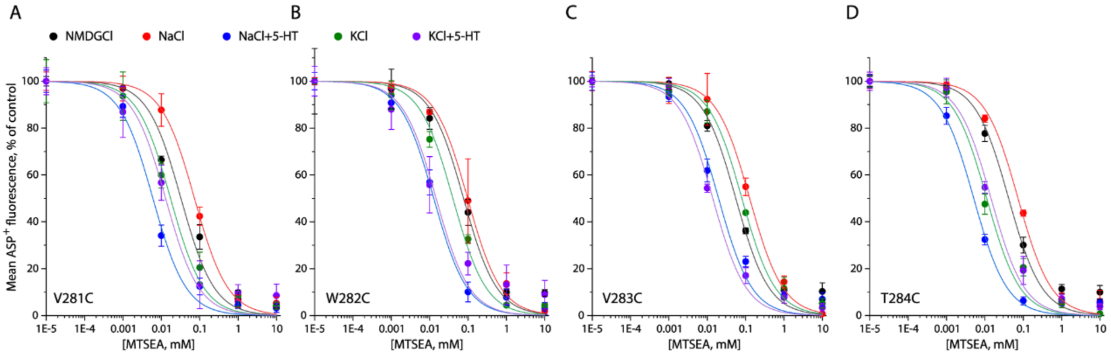

Figure S1. MTSEA concentration-dependent inhibition of ASP+ binding by cysteine mutants in the middle of TM5. Inhibition of ASP+ binding by cells stably expressing V281C/X5C (**A**), W282C/X5C (**B**), V283C/X5C (**C**), or T284C/X5C (**D**) was examined by incubating with MTSEA at a range of concentrations in HEPES buffer containing the indicated ion and substrate, as described in Section 4. The graphs show representative experiments for individual mutants. $n = 3$.

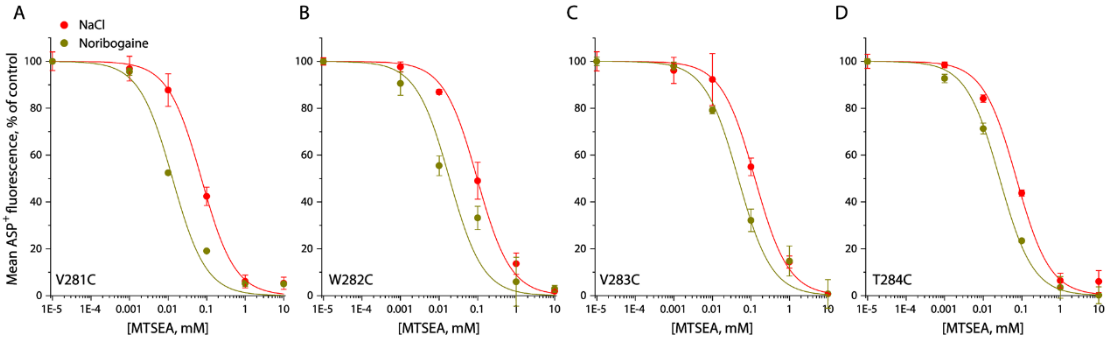

Figure S2. Effects of noribogaine binding on accessibility of cysteine mutants in the middle of TM5. Inhibition of ASP+ binding by cells stably expressing V281C/X5C (**A**), W282C/X5C (**B**), V283C/X5C (**C**), or T284C/X5C (**D**) was examined by incubating with MTSEA at a range of concentrations in HEPES buffer containing 150 mM NaCl in the presence or absence of 10 μM noribogaine, as described in Section 4. The graphs show representative experiments for individual mutants. $n = 3$.

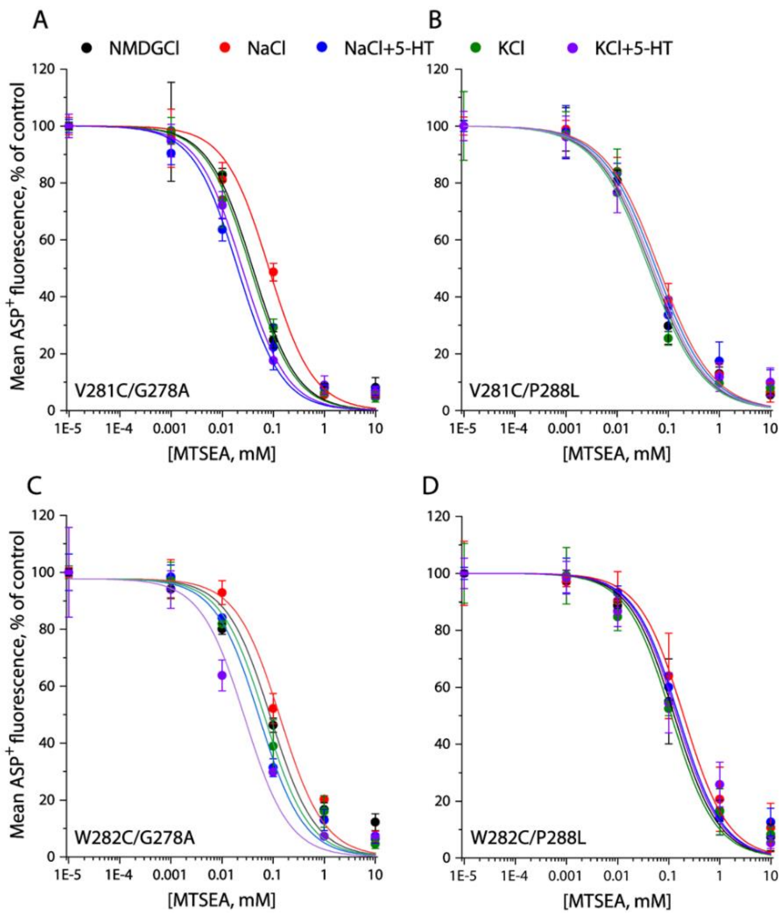

Figure S3. Effects of the GX_9P mutations on accessibility of cysteine mutants in the middle of TM5. Inhibition of ASP$^+$ binding by cells stably expressing V281C/G278A/X5C (**A**), V281C/P288L/X5C (**B**), W282C/G278A/X5C (**C**), or W282C/P288L/X5C (**D**) was examined by incubation with MTSEA at a range of concentrations in HEPES buffer containing the indicated ion and substrate, as described in Section 4. The graphs show representative experiments for individual mutants. $n = 3$.

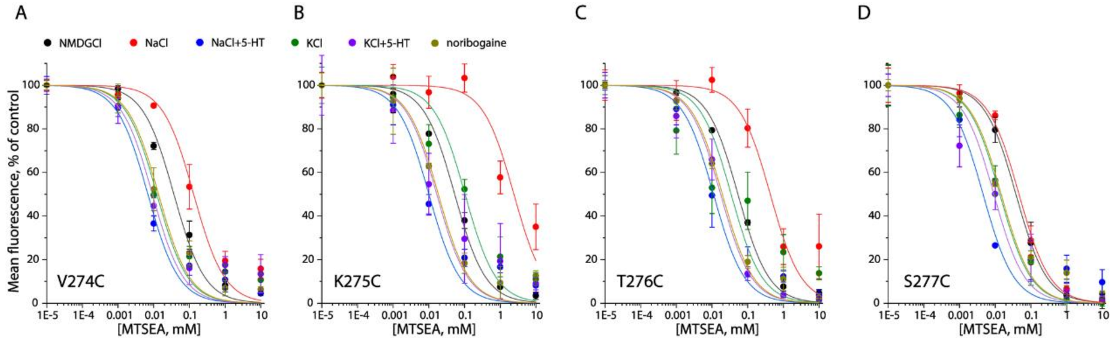

Figure S4. MTSEA concentration-dependent inhibition of ASP$^+$ binding by cysteine mutants in the intracellular end of TM5. Inhibition of ASP$^+$ binding by the cells stably expressing V274C/X5C (**A**), K275C/X5C (**B**), T276C/X5C (**C**), or S277C/X5C (**D**) was examined by incubating with MTSEA at a range of concentrations in HEPES buffer containing the indicated ion and substrate, as described in Section 4. The graphs show representative experiments for individual mutants. $n = 3$.

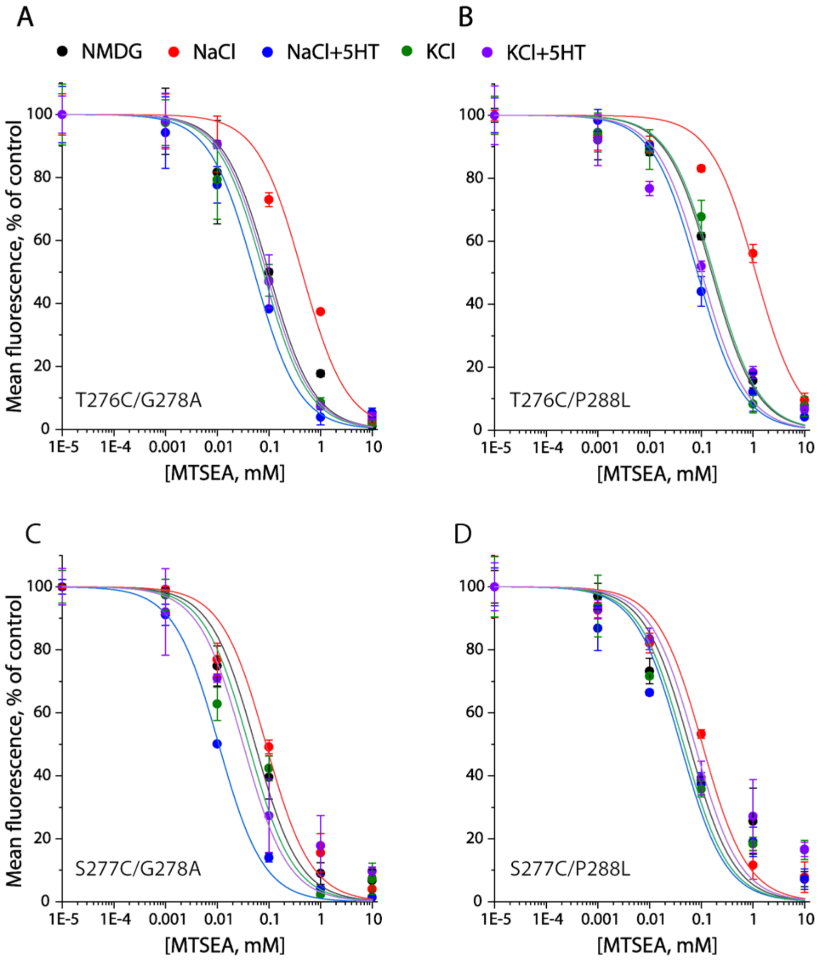

Figure S5. Effects of the GX₉P mutations on accessibility of cysteine mutants in the intracellular end of TM5. Inhibition of ASP+ binding by cells stably expressing T276C/G278A/X5C (**A**), T276C/P288L/X5C (**B**), S277C/G278A/X5C (**C**), or S277C/P288L/X5C (**D**) was examined by incubation with MTSEA at a range of concentrations in HEPES buffer containing the indicated ion and substrate, as described in Section 4. The graphs show representative experiments for individual mutants. $n = 3$.
